# Cutaneous adverse events associated with BRAF and MEK inhibitors: a systematic review and meta-analysis

**DOI:** 10.3389/fphar.2024.1457226

**Published:** 2024-12-24

**Authors:** Junhui Qian, Jinlong Wan, Qin Yao, Yin Chen, Tao Ling, Yuejuan Zhang, Zhihua Tang

**Affiliations:** ^1^ Department of Pharmacy, Shaoxing People’s Hospital, Shaoxing, China; ^2^ Department of Gastroenterology, Gaozhou People’s Hospital, Maoming, China; ^3^ Department of Pharmacy, Suqian First Hospital, Suqian, China

**Keywords:** cutaneous adverse events, BRAF inhibitor, mek inhibitor, melanoma, meta analysis

## Abstract

**Aim:**

Cutaneous adverse events (CAEs) after treatment with BRAF and MEK inhibitors in patients with melanoma remain incompletely characterized. To determine the association of BRAF and MEK inhibitor treatment with CAEs in patients with melanoma compared with BRAF inhibitor alone.

**Method:**

PubMed, Cochrane, Embase and Web of Science were systematically searched for BRAF and MEK inhibitors from database inception through 10 May 2024. Randomized clinical trials reporting on CAEs in patients with melanoma being treated with BRAF and MEK inhibitors compared with patients with melanoma being treated with BRAF inhibitor monotherapy were selected. Pooled Risk ratios (RRs) and 95% CIs were determined using random-effects analyses. The selected end points were alopecia, cutaneous squamous-cell carcinoma, hyperkeratosis, keratoacanthoma, palmoplantar erythrodysaesthesia syndrome, palmoplantar keratoderma, rash, photosensitivity reaction, and skin papilloma. All-grade and high-grade (≥3) CAEs were recorded.

**Results:**

Comparing with BRAF and MEK inhibitors, treatment with BRAF inhibitors alone was associated with an increased risk of rash (RR, 0.73; 95% CI, 0.54–0.99; *p* = 0.039; I^2^ = 88%), alopecia (RR, 0.28; 95% CI, 0.20–0.41; P < 0.001; I^2^ = 76%), hyperkeratosis (RR, 0.30; 95% CI, 0.22–0.41; P < 0.001; I^2^ = 56%), palmoplantar erythrodysaesthesia syndrome (RR, 0.21; 95% CI, 0.10–0.47; P < 0.001; I^2^ = 81%), palmoplantar keratoderma (RR, 0.39; 95% CI, 0.26–0.57; P < 0.001; I^2^ = 29%), Skin papilloma (RR, 0.25; 95% CI, 0.12–0.52; P < 0.001; I^2^ = 77%), cutaneous squamous-cell carcinoma (RR, 0.21; 95% CI, 0.11–0.42; P < 0.001; I^2^ = 50%), and keratoacanthoma (RR, 0.22; 95% CI, 0.12–0.40; P < 0.001; I^2^ = 0%).

**Conclusion:**

Therapy with BRAF and MEK inhibitors was associated with a lower risk of CAEs, especially rash, alopecia, hyperkeratosis, palmoplantar erythrodysaesthesia syndrome, palmoplantar keratoderma, skin papilloma, cutaneous squamous-cell carcinoma, and keratoacanthoma, compared with BRAF inhibitor alone. The risks of photosensitivity reaction was similar between the assessed groups. The findings may help to balance between beneficial melanoma treatment and cutaneous morbidity and mortality.

## Introduction

In the Western population, 1 out of every 50 individuals will develop melanoma (Dzwierzynski, 2021). The incidence of melanoma is increasing faster than any other malignancy, and melanoma remains an important challenge to cancer control and public health globally, especially in fair-skinned populations of European descent (Arnold et al., 2022). BRAF mutations are most frequent in patients with melanoma where they occur in approximately 50% of patients with advanced disease (Yélamos et al., 2016; Davies et al., 2002). The combination of the BRAF inhibitor and MEK inhibitor showed outstanding response rates in BRAF-mutated melanoma and is now considered the standard of care in this setting (Califano et al., 2024). Combined BRAF and MEK inhibitors, as compared with BRAF inhibitors alone, should be delay the emergence of resistance and should be reduce toxic effects in patients who have melanoma with BRAF V600E or V600K mutations (Long et al., 2014).

Combined BRAF and MEK inhibitors therapy has emerged as an optimal treatment of metastatic BRAF-mutated melanoma, with improved survival rates compared with BRAF inhibitors alone (Dummer et al., 2022; Ascierto et al., 2020). Multiple studies (Hauschild et al., 2018; Long et al., 2018; Planchard et al., 2016) found that the first combined BRAF and MEK inhibitors therapy showed significant improvement in an investigator-assessed overall response and relapse-free survival in treating melanoma, which led to global approval. Up to now, 3 BRAF inhibitors [dabrafenib (Hauschild et al., 2012), vemurafenib (Chapman et al., 2011), and encorafenib (Dummer et al., 2018a)] and 3 MEK inhibitors [trametinib (Flaherty et al., 2012a), cobinimetinib (Larkin et al., 2014), and binimetinib (Ascierto et al., 2013)] have received US Food and Drug Administration and European Medicines Agency approval.

Skin manifestations are increasingly documented with anticancer drugs, which are not uncommon, and can constitute a major challenge in clinical decision making (Raschi et al., 2022; Zhao et al., 2022). Prompt recognition and multidisciplinary management are critical to prevent unnecessary discontinuation or balance timely treatment interruption with early resumption to avoid cancer recurrence or progression (Barrios et al., 2020). BRAF inhibitor with or without MEK inhibitor have been associated with dermatologic reactions, especially rash, in pivotal trials. The nature and incidence of CAEs associated with combined BRAF and MEK inhibitors therapy are incompletely described. However, cutaneous complications may affect a patient’s quality of life or may require temporary or permanent cancer therapy termination. We set out to clarify the type, incidence, and Risk ratio of CAEs in patients with melanoma who are being treated with combined BRAF and MEK inhibitors therapy compared with patients receiving BRAF inhibitor alone in this systematic review and meta-analysis.

## Methods

Our systematic review and meta-analysis were based on the preferred reporting items for systematic review and meta-analysis (PRISMA) guidelines (Liberati et al., 2009) and were registered in PROSPERO (CRD42024553642).

A systematic search was conducted through PubMed, Cochrane, Embase and Web of Science databases, and abstracts or presentations from annual meetings of the major cutaneous and cancer societies to identify relevant studies published from the inception of the databases to 10 May 2024, using the search terms BRAF inhibitors (dabrafenib, vemurafenib, and encorafenib) and MEK inhibitors (trametinib, cobinimetinib, and binimetinib). We considered studies published in English only. The search strategy and results of all databases are depicted in the Supplementary material. Because the purpose of this study was to summarize the incidence of overall and high-grade CAEs in patients with melanoma receiving BRAF and MEK inhibitors, we restricted our study to randomized clinical trials (RCTs) in which adult participants received the available combinations of BRAF inhibitors and MEK inhibitors (ie, dabrafenib and trametinib, vemurafenib and cobimetinib, or encorafenib and binimetinib) and were randomly assigned to a treatment or a control group. The meta-analysis excluded abstracts, reviews, animal and *in vitro* studies, meta-analyses, case reports, single-arm BRAF and MEK inhibitor treatment studies, monotherapy with BRAF inhibitor studies, studies with MEK inhibitor treatment with other therapies for melanoma, nonrandomized clinical trials, studies that did not report on CAEs, and special population studies (e.g., elderly population, population from a certain geographic region, pediatric population). After removing duplicates, Junhui Qian and Zhihua Tang independently reviewed the abstracts. Any discrepancies in results between the 2 investigators were solved by discussion with the other investigators (Tao Ling). When the inclusion criteria appeared to be met, the full-text publication was reviewed by the 3 authors mentioned above. At the end of the review process, the full texts of the studies considered eligible were reviewed by all investigators.

### Data extraction and quality assessment

Junhui Qian and Zhihua Tang independently performed data extraction using a standard data extraction form that contained the following fields: (1) publication details (i.e., name of the first author and year of publication), (2) clinicalTrials.gov number, (3) study design, (4) characteristics of study population (i.e., cancer type, sample size, age, sex distribution, and ECOG PS), (5) treatment, and (6) mean follow-up.

The trial quality was assessed by Junhui Qian and Zhihua Tang for each study separately against the following criteria according to the Cochrane Risk-of-Bias Tool (Higgins and Green, 2011): (1) random sequence generation (i.e., selection bias), (2) allocation concealment (i.e., selection bias), (3) blinding of participants and personnel (ie, performance bias), (4) blinding of outcome assessment (i.e., detection bias), (5) incomplete outcome data, (6) selective reporting (i.e., reporting bias), and (7) other bias (i.e., measurement error, observer variability, dose of drug, length of follow-up, and characteristics of participants). Authors resolved disagreement by consensus, and a third author (Tao Ling) was consulted to resolve disagreement.

### Study end points

The end points were defined according to the National Cancer Institute Common Terminology Criteria for Adverse Events version 4. All-grade and high-grade (i.e., grade 3–5, indicating severe, life threatening, or causing death) treatment-emergent CAEs were abstracted. The selected end points were as follows: (1) alopecia, (2) cutaneous squamous-cell carcinoma, (3) hyperkeratosis, (4) keratoacanthoma, (5) palmoplantar erythrodysaesthesia syndrome, (6) palmoplantar keratoderma, (7) rash, (8) photosensitivity reaction, and (9) skin papilloma.

### Statistical analysis

The meta-analysis was conducted on eligible studies by dividing the patients into the following 2 groups: (1) the BRAF and MEK inhibitors group, which included patients with melanoma treated with a combination of BRAF inhibitors and MEK inhibitors; and (2) the control group, which included patients with melanoma treated with BRAF inhibitor alone. The proportion of patients with CAEs receiving BRAF and MEK inhibitors was compared with that of the control group in the same RCT. The data are expressed as percentage of patients with CAEs, calculated by dividing the number of each CAE by the total sample size. Risk ratios (RRs) and 95% CIs are used to express dichotomous outcomes (Viera, 2008). Statistical significance was set at *p* < 0.05, and all tests were 2-tailed. A 2012 study (Flaherty et al., 2012b) had 3 arms: combination therapy with dabrafenib (150 mg) plus trametinib (1 or 2 mg) or dabrafenib monotherapy. A 2018 study (Dummer et al., 2018b) had 3 arms: either encorafenib plus binimetinib (encorafenib plus binimetinib group), encorafenib (encorafenib group), or vemurafenib (vemurafenib group).

For the analysis, we used random-effects. A random-effects model was preferred owing to the assumption that different studies are estimating different yet related intervention effects. In the presence of heterogeneity, the use of the random-effects method will result in wider CIs for the average intervention and corresponding claims of statistical significance will be more conservative (Higgins and Green, 2011). Statistical heterogeneity was reported as the Q statistic and I^2^ statistics. A value of I^2^ less than 40% denoted that heterogeneity might not be important, I^2^ from 40% to 60% may have represented moderate heterogeneity, I^2^ from 50% to 90% may have represented substantial heterogeneity, and I^2^ from75% to 100% represented considerable heterogeneity (Higgins and Green, 2011). The funnel plot test could not be used to assess publication bias because our analysis included fewer than 10 studies (Higgins and Green, 2011). The analyses were conducted using R 4.4.0 and Revman 5.3.5.

## Results

### Eligible studies and characteristics

**FIGURE 1:**
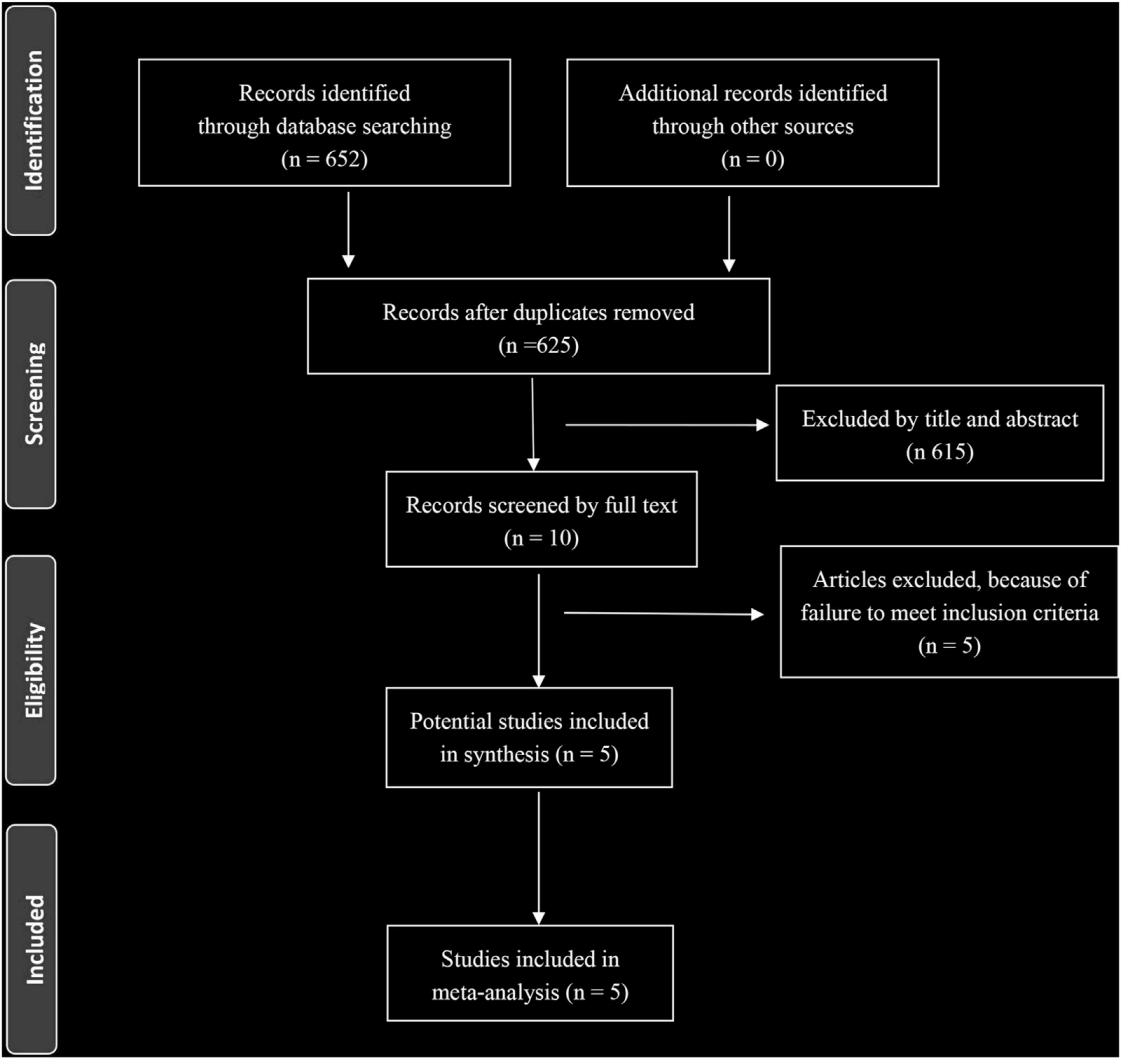
Prisma selection flowchart.

A total of 652 eligible articles were identified through database searching (Figure 1). We identified 5 RCTs of patients receiving BRAF and MEK inhibitors therapy compared with patients receiving BRAF inhibitor alone (Flaherty et al., 2012b; Dummer et al., 2018b; Robert et al., 2015; Ascierto et al., 2016; Long et al., 2017). A total of 2,361 patients with melanoma were included. General characteristics of the study population are detailed in Table 1.

**TABLE 1:** Characteristics of the studies.

Study	ClinicalTrials.gov number	Type	Cancer type	ECOG PS = 0, *n* (%)	Treatment	*n*. Of patients	Age, mean (range), y	Male, *n* (%)	Follow-up, median (IQR), mo
Flaherty et al. (2012b)	NCT01072175	RCT II	Metastatic melanoma and BRAF V600 mutations	38 (70)	Dabrafenib 150 mg twice daily and trametinib 1 mg once daily	54	49 (23–85)	30 (56)	14.1 (10.8–17.6)
				35 (65)	Dabrafenib 150 mg twice daily and trametinib 2 mg once daily	54	58 (27–79)	34 (63)	
				34 (63)	Dabrafenib 150 mg twice daily	54	50 (18–82)	29 (54)	
Robert et al. (2015)	NCT01597908	RCT III	Unresectable stage IIIC or IV melanoma with BRAF V600 mutations	248 (71)	Dabrafenib 150 mg twice daily and trametinib 2 mg once daily	352	55 (18–91)	208 (59)	10 (NA)
				248 (70)	Vemurafenib 960 mg twice daily	352	54 (18–88)	180 (51)	11 (NA)
Ascierto et al. (2016)	NCT01689519	RCT III	Unresectable stage IIIC or stage IV melanoma with BRAF V600 mutations	184 (76)	Vemurafenib 960 mg twice daily and cobimetinib 60 mg once daily	247	56 (23–88)	146 (59%)	14.2 (8.5–17.3)
				164 (67)	Vemurafenib 960 mg twice daily	248	55 (25–85)	140 (56%)	>36 (NA)
Long et al. (2017)	NCT01584648	RCT III	Unresectable stage IIIC or stage IV melanoma with BRAF V600 mutation	155 (73)	Dabrafenib 150 mg twice daily and trametinib 2 mg once daily	211	55 (22–89)	111 (53)	>36 (NA)
				150 (71)	Dabrafenib 150 mg twice daily	212	57 (22–86)	114 (54)	
Dummer et al. (2018b)	NCT01909453	RCT III	Unresectable stage stage IIIB, IIIC, or IV, with BRAF V600 mutations	136 (71)	Encorafenib 450 mg once daily and binimetinib 45 mg twice daily	192	57 (20–89)	115 (60%)	16.6 (14.8–16.9)
				140 (72)	Encorafenib 300 mg once daily	194	54 (23–88)	108 (56%)	
				140 (73)	Vemurafenib 960 mg twice daily	191	54 (23–88)	111 (58%)	

### Risk ratios of all-grade CAEs

**FIGURE 2:**
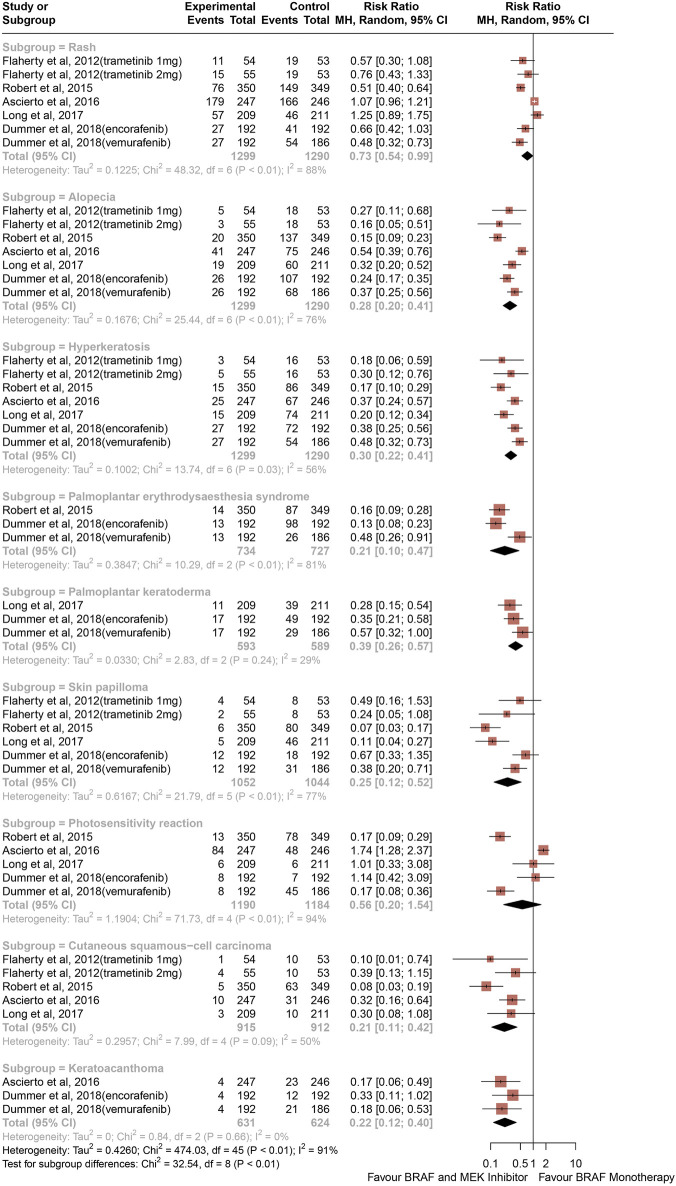
Overall study estimates of the risk ratio (RR) of cutaneous adverse events of all grades associated with BRAF and MEK inhibitor treatment vs. BRAF inhibitor monotherapy.

The risk of all-grade CAEs calculated as RRs are depicted in Figure 2. Comparing with BRAF and MEK inhibitors, treatment with BRAF inhibitors alone was associated with an increased risk of rash (RR, 0.73; 95% CI, 0.54–0.99; *p* = 0.039; I^2^ = 88%), alopecia (RR, 0.28; 95% CI, 0.20–0.41; P < 0.001; I^2^ = 76%), hyperkeratosis (RR, 0.30; 95% CI, 0.22–0.41; P < 0.001; I^2^ = 56%), palmoplantar erythrodysaesthesia syndrome (RR, 0.21; 95% CI, 0.10–0.47; P < 0.001; I^2^ = 81%), palmoplantar keratoderma (RR, 0.39; 95% CI, 0.26–0.57; P < 0.001; I^2^ = 29%), skin papilloma (RR, 0.25; 95% CI, 0.12–0.52; P < 0.001; I^2^ = 77%), cutaneous squamous-cell carcinoma (RR, 0.21; 95% CI, 0.11–0.42; P < 0.001; I^2^ = 50%), and keratoacanthoma (RR, 0.22; 95% CI, 0.12–0.40; P < 0.001; I^2^ = 0%) (Figure 2). The RR of photosensitivity reaction were similar between the BRAF and MEK inhibitor group and the control group (RR, 0.56; 95% CI, 0.20–1.54; *p* = 0.258; I^2^ = 94%) (Figure 2).

Overall, 32.97% of patients from the BRAF and MEK inhibitor treatment group had risk of rash compared with 38.40% in the control group, and 10.3% of patients in the BRAF and MEK treatment group developed alopecia, compared with 37.59% in the BRAF inhibitor control group. Analysis revealed that therapy with BRAF and MEK inhibitors was associated with lower risk of all-grade hyperkeratosis than BRAF inhibitor alone, with 8.13% of patients from the BRAF and MEK inhibitors therapy group developing hyperkeratosis compared with 29.83% of the BRAF inhibitor alone group. The proportion of patients from the BRAF inhibitor alone treatment group who had a high-grade CAE compared with the BRAF and MEK inhibitor treatment group were 29.02% vs. 4.98% for palmoplantar erythrodysaesthesia syndrome and 19.86% vs. 6.98% for palmoplantar keratoderma. 3.37% of patients from the BRAF and MEK inhibitor treatment group had a decrease in skin papilloma compared with 18.47% in BRAF inhibitor alone group. BRAF inhibitor alone was associated with a 5.2-fold increase in risk of cutaneous squamous-cell carcinoma (13.27% vs. 2.51%) and a higher risk keratoacanthoma (8.97% vs. 1.82%) than the combination of BRAF and MEK inhibitors.

### Risk ratios of high-grade CAEs

**FIGURE 3:**
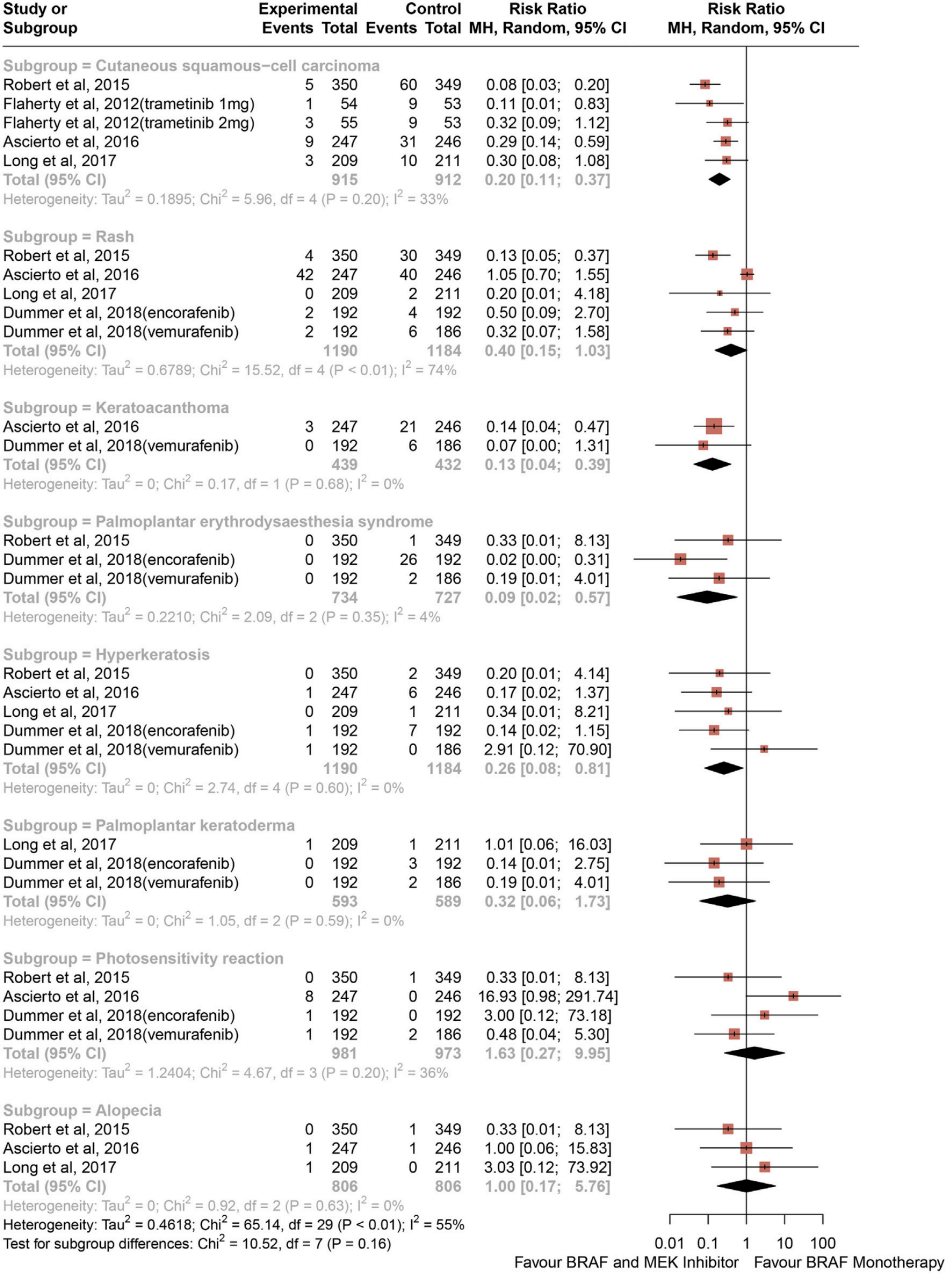
Overall study estimates of the risk ratio (RR) of cutaneous adverse events of Grade 3 or 4 associated with BRAF and MEK inhibitor Treatment vs. BRAF inhibitor monotherapy.

The risk of high-grade CAEs calculated as RRs are depicted in Figure 3. Comparing with BRAF and MEK inhibitors, treatment with BRAF inhibitors alone was associated with an increased risk of hyperkeratosis (RR, 0.26; 95% CI, 0.08–0.81; *p* = 0.021; I^2^ = 0%), palmoplantar erythrodysaesthesia syndrome (RR, 0.09; 95% CI, 0.02–0.57; *p* = 0.010; I^2^ = 4%), cutaneous squamous-cell carcinoma (RR, 0.20; 95% CI, 0.11–0.37; P < 0.001; I^2^ = 33%), and keratoacanthoma (RR, 0.13; 95% CI, 0.04–0.39; P < 0.001; I^2^ = 0%) (Figure 3). In contrast with the risk of all-grade rash, alopecia, and palmoplantar keratoderma, the risk of high-grade rash, alopecia, palmoplantar keratoderma, and photosensitivity reaction were similar between groups (RR, 0.40; 95% CI, 0.15–1.03; *p* = 0.057; I^2^ = 74%) (RR, 1.00; 95% CI, 0.17–5.76; *p* = 0.999; I^2^ = 0%) (RR, 0.32; 95% CI, 0.06–1.73; *p* = 0.188; I^2^ = 0%) (RR, 1.63; 95% CI, 0.27–9.95; *p* = 0.594; I^2^ = 36%), respectively, (Figure 3).

Overall, 0.20% of patients from the BRAF and MEK inhibitor treatment group had a decrease in hyperkeratosis compared with 1.35% in the control group, and 0.00% of patients in the BRAF and MEK treatment group developed palmoplantar erythrodysaesthesia syndrome, compared with 3.99% in the BRAF inhibitor control group. The proportion of patients from the BRAF inhibitor alone treatment group who had a high-grade CAE compared with the BRAF and MEK inhibitor treatment group were 12.81% vs. 2.30% for cutaneous squamous-cell carcinoma and 6.25% vs. 0.68% for keratoacanthoma.

### Heterogeneity and sensitivity analysis

The heterogeneity for each analysis of all-grade CAEs was statistically significant, except for photosensitivity reaction analyses, where heterogeneity could be rated as substantial. The heterogeneity for each analysis of high-grade CAEs was statistically significant, except for alopecia, palmoplantar keratoderma, photosensitivity reaction, and rash analyses, where heterogeneity could be rated as low.

A sensitivity analysis was performed by excluding each study in a stepwise manner from the analysis to determine the relative importance of each study. Treatment with BRAF inhibitor alone remained a risk factor for the selected outcomes.

### Publication bias assessment

**FIGURE 4:**
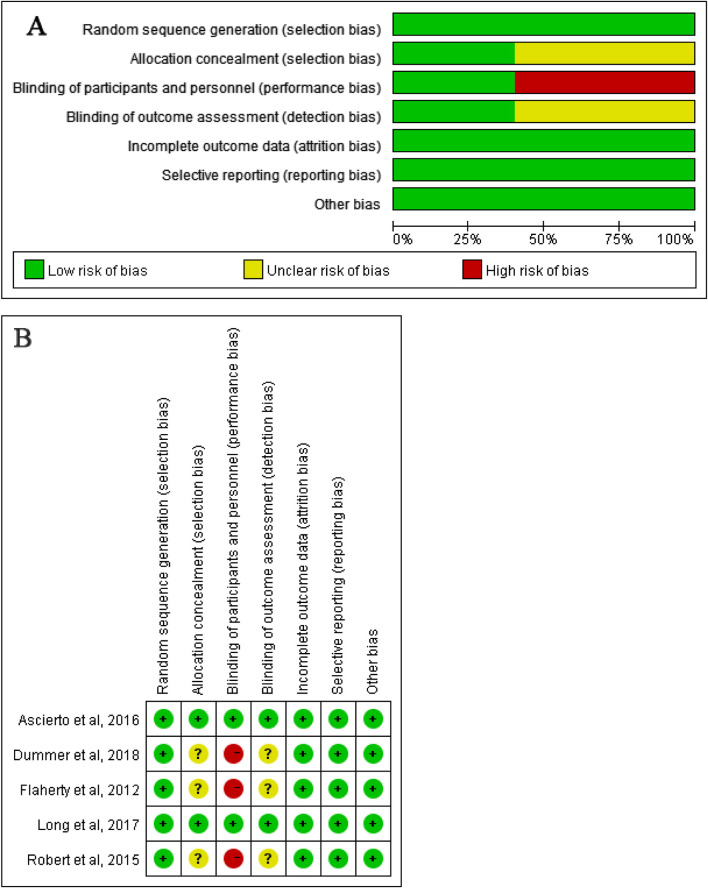
The Quality of the included studies **(A)** Risk of bias graph; **(B)** Risk of bias summary).

The studies were reviewed for publication bias. The risk of bias of the included studies was depicted in Figure 4.

## Discussion

Up to now, this is the first systematic review and meta-analysis pooled 5 RCTs with 2,361 participants to present a relatively comprehensive overview of the association between CAEs in patients treated with combined BRAF and MEK inhibitors therapy or patients receiving BRAF inhibitor alone. Common combination therapies are dabrafenib and trametinib, vemurafenib and cobimetinib, and encorafenib and binimetinib. BRAF inhibitor monotherapy are dabrafenib, vemurafenib, and encorafenib. To our knowledge, this is the first study to calculate the RRs of alopecia, cutaneous squamous-cell carcinoma, hyperkeratosis, keratoacanthoma, palmoplantar erythrodysaesthesia syndrome, palmoplantar keratoderma, rash, photosensitivity reaction, and skin papilloma.

The main results of our study were as follows: (1) BRAF and MEK inhibitor therapy was associated with a lower RR of rash, alopecia, hyperkeratosis, palmoplantar erythrodysaesthesia syndrome, palmoplantar keratoderma, skin papilloma, cutaneous squamous-cell carcinoma, and keratoacanthoma compared with BRAF inhibitor monotherapy; (2) The RR of photosensitivity reaction were similar between the BRAF and MEK inhibitor therapy and BRAF inhibitor monotherapy; (3) the RRs of high-grade hyperkeratosis, palmoplantar erythrodysaesthesia syndrome, cutaneous squamous-cell carcinoma, and keratoacanthoma were lower in the group being treated with BRAF and MEK inhibitors than in the group being treated with BRAF inhibitor monotherapy. (4) In contrast with the risk of all-grade rash, alopecia, and palmoplantar keratoderma, the risk of high-grade rash, alopecia, palmoplantar keratoderma, and photosensitivity reaction were similar between the BRAF and MEK inhibitor therapy and BRAF inhibitor monotherapy.

It is interesting to note that when applying MEK inhibitors with BRAF inhibitor can reduce cutaneous toxicities, the effects that are mediated by suppression cause skin toxicity reaction of special activation of MAP kinase pathways. Cutaneous toxicities, most notably squamous cell carcinomas (SCC), are considered a mechanism-related class effect of BRAF inhibitors. The development of cutaneous toxicities of BRAF inhibitors may be explained by paradoxical activation of the MAPK pathway in wild-type BRAF cells. The formation of homo or hetero RAF dimers in wild-type BRAF cells in the presence of oncogenic RAS mutation and subsequently activation of MEK is considered the major cause for the observed cutaneous adverse effects of BRAF inhibitor (Heidorn et al., 2010; Hatzivassiliou et al., 2010). The mechanism is not only suggested by preclinical studies, it is also demonstrated by the high prevalence of oncogenic RAS mutation in clinical samples for patients who developed SCC with BRAF inhibitor treatment (Su et al., 2012; Anforth et al., 2012). Concomitant MEK inhibitor administration has improved the skin toxicity profile of BRAF inhibitor by multiple clinical studies (Robert et al., 2015; Ribas et al., 2014). Sanlorenzo et al. directly performed a retrospective cohort study, collecting data from 44 melanoma patients treated either with BRAF inhibitors (vemurafenib or dabrafenib) or BRAF and MEK inhibitor combination regimens (vemurafenib + cobimetinib or dabrafenib + trametinib) (Sanlorenzo et al., 2014). As expected, cutaneous AEs were less frequent in patients with BRAF inhibitor and MEK inhibitor combination compared with those in patients with BRAF inhibitor alone, and cutaneous AEs occurred more frequently and faster during BRAF inhibitor therapy than during BRAF and MEK inhibitors combination therapy among patients who received single treatment regimen (either BRAF inhibitor monotherapy or BRAF and MEK inhibitors combination treatment). In particular, they observed a longer cutaneous adverse event-free interval during treatment with a combination of dabrafenib and trametinib. Treatment with vemurafenib causes a multitude of cutaneous AEs, such as exanthema, photosensitivity, palmarplantar dysesthesia or hand-foot syndrome, alopecia, pruritus, hyperkeratosis, skin papillomas, keratoacanthomas and cutaneous squamouscell carcinomas (Chapman et al., 2011; Flaherty et al., 2012b; Mattei et al., 2013). The most frequent cutaneous AEs of dabrafenib are hyperkeratosis, papilloma, alopecia, and palmar-plantar erythrodysesthesia syndrome. Trametinib is more frequently related with the development of acneiform dermatitis or alopecia (Flaherty et al., 2012a; Anforth et al., 2014). Less is known about the cutaneous AEs related to cobimetinib.

In our study, 32.97% of patients from the BRAF and MEK inhibitor treatment group had a decrease in rash compared with 38.40% in the BRAF inhibitor alone control group. Common AEs reported at a lower frequency (with a difference in proportion of patients of 10% or higher) in the encorafenib plus binimetinib group than in the encorafenib or vemurafenib groups were toxic effects to the skin (e.g., pruritus, hyperkeratosis, rash, keratosis pilaris, palmoplantar keratoderma, palmoplantar erythrodysaesthesia syndrome, dry skin, skin papilloma, macropapular rash, and sunburn), alopecia, and photosensitivity reaction (Dummer et al., 2018b). In our study, 10.3% of patients in the BRAF and MEK treatment group developed alopecia, compared with 37.59% in the BRAF inhibitor alone control group. In one study, the most frequent cutaneous AEs were rash (43%), alopecia (39%) in the vemurafenib group (Robert et al., 2015). Cutaneous effects were more frequent in the vemurafenib group than in the combination-therapy group, in particular rash (43% vs. 22%), photosensitivity reaction (22% vs. 4%), hand-foot syndrome (25% vs. 4%), skin papillomas (23% vs. 2%), squamous-cell carcinomas and keratoacanthomas (18% vs. 1%), and hyperkeratosis (25% vs. 4%) (Robert et al., 2015). Analysis revealed that therapy with BRAF and MEK inhibitors was associated with lower risk of all-grade hyperkeratosis than BRAF inhibitor alone, with 8.13% of patients from the BRAF and MEK inhibitors therapy group developing hyperkeratosis compared with 29.83% of the BRAF inhibitor alone group. The development of hyperkeratosis, a well-known precursor of cutaneous squamous cell carcinomas, was frequent during monotherapy with both BRAF inhibitors. It has be reported that the development of cutaneous squamous cell carcinomas during BRAF inhibitor therapy is caused by activation of the MAPK pathway in keratinocytes with preexisting RAS mutations commonly found in chronically sun damaged skin. The proportion of patients from the BRAF inhibitor alone treatment group who had a high-grade CAEs compared with the BRAF and MEK inhibitor treatment group were 29.02% vs. 4.98% for palmoplantar erythrodysaesthesia syndrome and 19.86% vs. 6.98% for palmoplantar keratoderma. Photosensitivity is another well-known AE experienced during vemurafenib treatment (Chapman et al., 2011; Sosman et al., 2012). Previous studies speculated that this is due to the chemical structure of the drug and ultraviolet A exposure, rather than due to BRAF inhibitor and the subsequent consequences on MAPK signaling (Dummer et al., 2012). In our experience, also, photosensitivity was more frequent in patients treated with vemurafenib. Regardless of the treatment regimen, anytime a patient receives vemurafenib, particular attention should be given to sun exposure prevention measures. From the results of this study, we conclude that clinical decision-making can be helped according to patients’ history of skin-related AEs. Physicians can preferentially choose BRAF and MEK inhibitors treatment, when facing patients with better economic conditions or similar skin toxicity in the past.

## Limitations

This study has some limitations that need to be addressed. First, the double therapy regimen was compared with single therapy, and a perfect delimitation of the adverse events deriving from BRAF inhibitors or MEK inhibitors cannot be done. Second, the treatment regimens were different between the studies; although from the same class of therapies, there are some specific adverse events related to each regimen. Third, 2 study (Flaherty et al., 2012b; Dummer et al., 2018b) were analyzed as 2 separate studies, which could induce bias in the final analysis. However, excluding an arm from the final analysis did not influence the conclusion.

## Conclusion

In conclusion, therapy with BRAF and MEK inhibitors was associated with an decreased risk of CAEs, especially rash, alopecia, hyperkeratosis, palmoplantar erythrodysaesthesia syndrome, palmoplantar keratoderma, skin papilloma, cutaneous squamous-cell carcinoma, and keratoacanthoma, compared with BRAF inhibitor monotherapy. The risks of photosensitivity reaction was similar between the assessed groups. These adverse events should be carefully approached in skin-oncology teams for an optimal treatment of patients with melanoma.
