# Supplementary material for: Cutaneous adverse events associated with BRAF and MEK inhibitors: a systematic review and meta-analysis
Source: Front Pharmacol. 2024 Dec 24;15:1457226. doi: 10.3389/fphar.2024.1457226 (PMC11703664; doi:10.3389/fphar.2024.1457226)
Supplement: Supplementary file 1 [file DataSheet1.docx]

**Supplementary Files**

**Supplementary file 1: Search strategy**

**1. PubMed**

**Access Date:** **10 May 2024**

**Search Results: 18**

Search strategy:

#1 Vemurafenib[Title/Abstract] OR Dabrafenib[Title/Abstract] OR Encorafenib[Title/Abstract] OR Trametinib[Title/Abstract] OR Binimetinib[Title/Abstract] OR Cobinimetinib[Title/Abstract]

#2 Melanomas[Title/Abstract] OR Malignant Melanoma[Title/Abstract] OR Malignant Melanomas[Title/Abstract] OR Melanoma, Malignant[Title/Abstract] OR Melanomas, Malignant[Title/Abstract]

#3 randomized controlled trial[Publication Type] OR randomized[Title/Abstract] OR placebo[Title/Abstract]

#4 #1 AND #2 AND #3

**2. Web of Science**

**Access Date:10 May 2024**

**Search Results: 543**

Search strategy:

#1 TS= (Vemurafenib OR Dabrafenib OR Encorafenib OR Trametinib OR Binimetinib OR Cobinimetinib)

#2 TS= (Melanomas OR Malignant Melanoma OR Malignant Melanomas OR Melanoma, Malignant OR Melanomas, Malignant)

#3 TS= (random*)

#4 #1 AND #2 AND #3

**3. Embase**

**Access Date: 10 May 2024**

**Search Results:31**

Search strategy:

#1 'Vemurafenib':ab,ti OR 'Dabrafenib':ab,ti OR 'Encorafenib':ab,ti OR 'Trametinib':ab,ti OR 'Binimetinib':ab,ti OR 'Cobinimetinib':ab,ti

#2 'Melanomas':ab,ti OR 'Malignant Melanoma':ab,ti OR 'Malignant Melanomas':ab,ti OR 'Melanoma, Malignant':ab,ti OR 'Melanomas, Malignant':ab,ti

#3 'random' OR 'placebo' OR 'double-blind'

#4 #1 AND #2 AND #3

**4. Cochrane Library**

**Access Date: 10 May 2024**

**Search Results: 60**

Search strategy:

#1 (Vemurafenib):ti,ab,kw OR (Dabrafenib):ti,ab,kw OR (Encorafenib):ti,ab,kw OR (Trametinib):ti,ab,kw OR (Binimetinib):ti,ab,kw OR (Cobinimetinib):ti,ab,kw

#2 (Melanomas):ti,ab,kw OR (Malignant Melanoma):ti,ab,kw OR (Malignant Melanomas):ti,ab,kw OR (Melanoma, Malignant):ti,ab,kw OR (Melanomas, Malignant):ti,ab,kw

#3 #1 AND #2
